# Amine Functionalization of Channels of Metal‐Organic Frameworks for Effective Chemical Fixation of Carbon Dioxide: A Comparative Study with Three Newly Designed Porous Networks

**DOI:** 10.1002/open.202400110

**Published:** 2024-05-13

**Authors:** Rajib Moi, Swati Bedi, Kumar Biradha

**Affiliations:** ^1^ Department of Chemistry Indian Institute of Technology Kharagpur 721302 Kharagpur India

**Keywords:** Metal-organic frameworks, carbon dioxide fixation, gas adsorption, heterogeneous catalyst, triazole

## Abstract

Catalytic transformation of CO_2_ into value‐added chemical products can provide an appropriate solution for the raising environmental issues. To date, various metal‐organic frameworks (MOFs) with transition metal ions have been explored for CO_2_ capture and conversion, but alkaline earth metal‐based MOFs are comparatively less studied. Metal ions like Sr(II) having relatively large radius give rise to a high coordination number resulting in higher stability of the MOFs. Moreover, the introduction of N‐rich functional group in organic linker like −NH_2_, −CONH− and triazole into MOF backbone enhance their CO_2_ capture and conversion efficiency. Herein, the effect of amine group on the catalytic efficiency of MOFs for CO_2_ cycloaddition with epoxides under solvent free and ambient conditions are presented. The di‐carboxylates, such as 5‐aminoisophthalate (**AmIP**) and 5‐bromoisophthalate (**BrIP**) were utilized to synthesize Sr(II) based MOFs. The Zn(II) MOF was synthesized using tetra‐carboxylate containing amide spacer (**OAT**) and 4‐amino‐4H‐1,2,4‐triazole (**AMT**). All three MOFs exhibited porous networks with guest available volume ranging from 15 to 58 %. The catalytic efficiency of the MOFs towards carbon dioxide fixation reaction was explored. The catalytic performances revealed that the presence of amine group in the channels enhances the catalytic efficiency of the MOFs.

## Graphical abstract

The effect of amine group on the catalytic efficiency of metal‐organic frameworks (MOFs) for CO_2_ cycloaddition with epoxides are studied with newly designed 3D functionalized MOFs. The catalytic performances were compared for the MOFs and the reusability of the synthesized MOFs were also investigated.

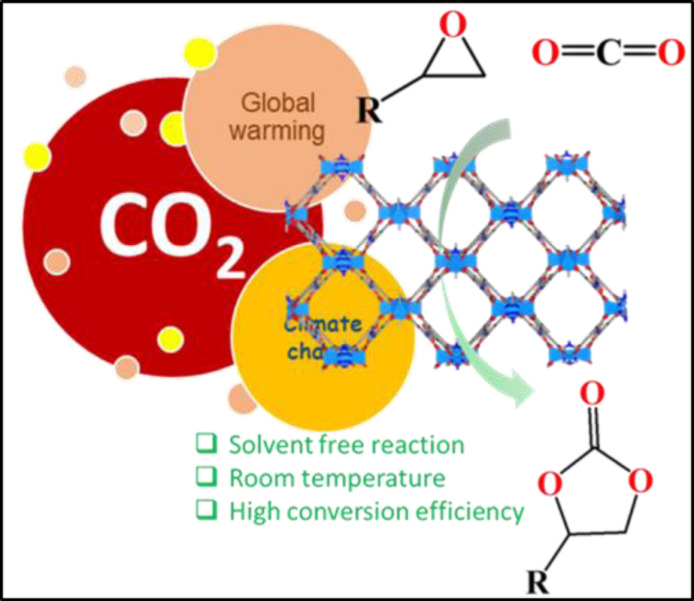

## Introduction

The development of carbon dioxide capture and sequestration (CCS) technologies has gained considerable attention given the serious global warming caused by excessive CO_2_ emissions due to modern day lifestyle, motorized tools, industries and utilization of fossil fuels.[^1^, ^2^, ^3^, ^4^, ^5^, ^6^, ^7^, ^8^] Catalytic transformation of the adsorbed CO_2_ into value‐added materials has been considered to be an appropriate solution to deal with the environmental issues and energy challenges.[^9^, ^10^, ^11^, ^12^] Various strategies have been developed to convert carbon dioxide into valuable chemicals. One of the green and atom economical strategy is cycloaddition of CO_2_ to an epoxide to produce cyclic carbonates without the formation of any byproduct. These cyclic carbonates are useful industrial raw materials in the manufacture of polymers and pharmaceuticals.[^13^, ^14^, ^15^] However, the kinetic inertness of carbon dioxide poses a severe problem to drive this reaction; therefore, efficient catalytic systems for its activation and conversion is a necessity and a hot topic of present‐day research.^[16]^ Materials like metal oxides,^[17]^ functionalized silica,^[18]^ and zeolites^[19]^ have been utilized to catalyze these reactions of CO_2_. The contemporary research suggests that the metal‐organic frameworks can be utilized as heterogeneous catalysts for the efficient reaction of CO_2_ with epoxides to produce corresponding cyclic carbonates under mild reaction conditions.

**Scheme 1:**
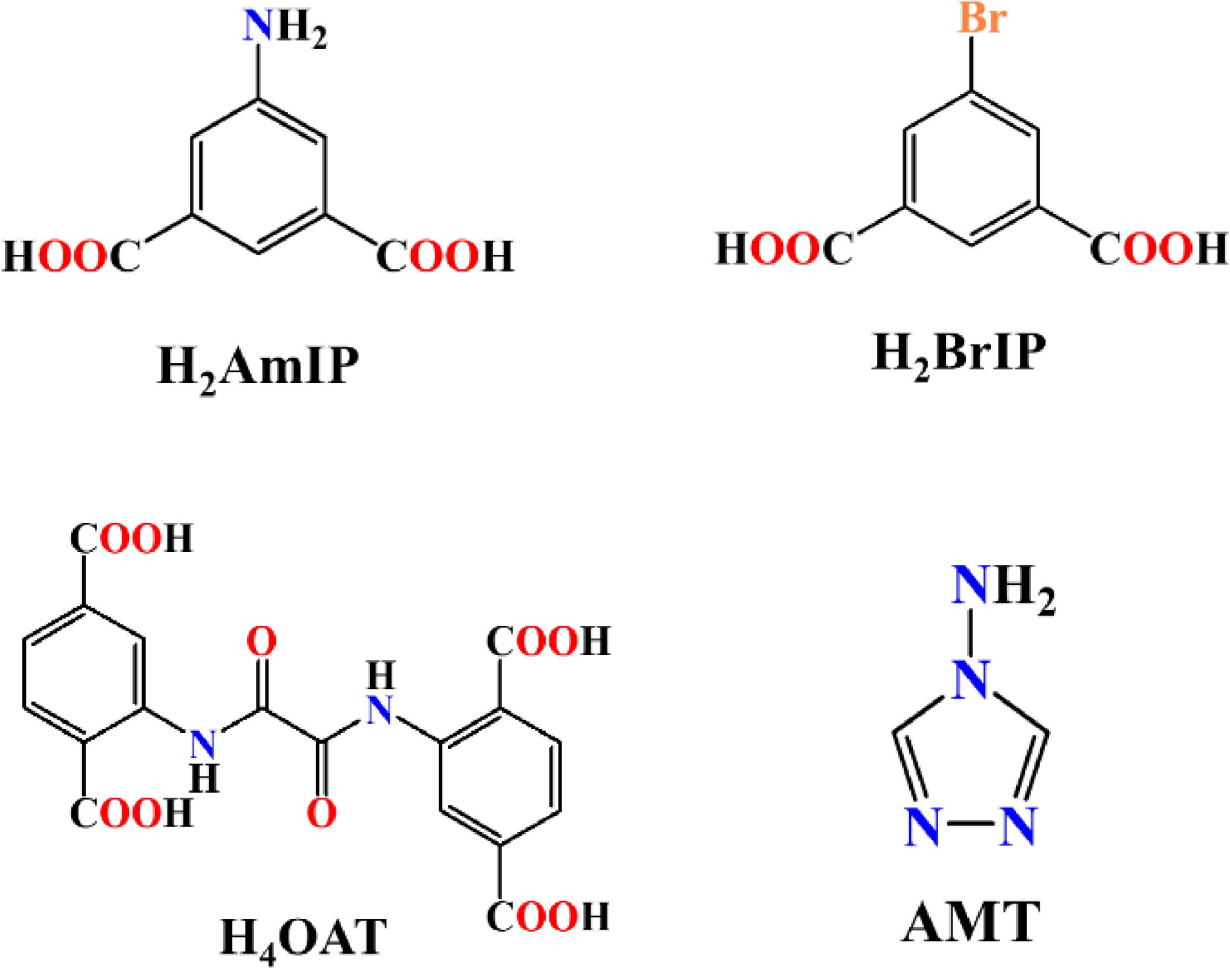
Structure of the molecules used in this study.

Metal‐organic frameworks are hybrid organic‐inorganic materials with widespread applications in various frontier domains of science and technology,^[20]^ such as gas adsorption and separation, chemical and biomedical sensors, magnetism, light harvesting and electrochemical energy conversion and storage.[^21^, ^22^, ^23^, ^24^, ^25^, ^26^] The surge in popularity of MOFs in such diverse areas can be attributed to their amenability to design a desired structure by judicious choice of the metal ion containing secondary building unit (SBU) and the organic linker.[^27^, ^28^] Among the huge range of applications, MOFs as catalysts are more desirable because of confinement effect and their inherent property of containing porous channels which can act as preconcentrator to improve the rate of the reaction.[^29^, ^30^] However, the adsorption of CO_2_ gas on the catalyst surface is challenging because carbon dioxide is a linear molecule (C−O: 1.16 Å) with zero dipole moment. However, it contains polar bonds due to electronegativity difference between C and O and a high quadrupole moment (3.4×10^−40^ cm^2^). The electronic structure of CO_2_ can be depicted as O^δ−^−C^2δ+^−O^δ−^ which shows susceptibility of central carbon atom for nucleophilic attack. Introducing N‐rich functional group containing ligands like −NH_2_ group, −CONH− groups and triazoles into MOFs is considered as very useful strategy to enhance the CO_2_ gas adsorption capacity.[^31^, ^32^, ^33^] This strategy has been explored in several MOFs, for example Long and co‐workers introduced ethylenediamine into Cu‐BTTri to obtain Cu‐BTTri‐en which shows higher CO_2_ adsorption (0.366 mmol/g) than original Cu‐BTTri (0.277 mmol/g).^[34]^ In another study, Palomino and co‐workers reported grafting of ethylenediamine onto MIL‐100(Cr) and the results showed higher CO_2_ adsorption capacity of 2.4 mmol g^−1^ as compared with 1.6 mmol g^−1^ for MIL‐100(Cr).^[35]^ In a recent report, Wang and co‐workers studied the amino triazole based Zn(II) MOF for high CO_2_ adsorption and higher efficiency for carbon dioxide fixation.^[36]^ From our group, the amide containing tetracarboxylate Cu‐MOFs have been reported for high CO_2_ adsorption and high catalytic activity for the cycloaddition of CO_2_ to the various epoxides.^[37]^ Further, Lewis acidic open metal sites are required for the catalytic conversion and choice of appropriate metal centre is crucial to obtain the desired catalytic performance of MOFs. Most commonly first‐row transition metal ions are well explored in this regard and alkaline earth metal ions are comparatively less explored.[^38^, ^39^] However, the high charge density of these metal ions led to ionic nature of the bond results in stronger bonding interactions with carboxylate oxygen. The alkaline earth metal ions like bivalent strontium have relatively larger radius that gives rise to a high coordination number resulting in higher stability of the MOFs.[^40^, ^41^, ^42^] Moreover, the strong Lewis acidity of alkaline earth metal ions also provides active sites for the heterogeneous catalysis.[^43^, ^44^, ^45^] However, Sr‐MOFs are less explored as heterogeneous catalyst for CO_2_ fixation and there are no reports showing the effect of ligand functionalization on the catalytic efficiency of Sr‐MOFs for this reaction.^[46]^ In this study, the effect of amine group on the catalytic efficiency of MOF for CO_2_ cycloaddition with epoxides are presented. 5‐aminoisophthalic acid and 5‐bromoisophthalic acid were utilized as functional organic linkers (Scheme 1) and Sr(II) ion for construction of the nodes to synthesize two new MOFs. A new Zn(II)‐MOF with 4‐amino‐4H‐1,2,4‐triazole and an amide based tetracarboxylate ligand was studied for its catalytic efficiency towards carbon dioxide fixation reaction in solvent free condition under ambient temperature and pressure. The catalytic performances of these three MOFs were compared and the reusability of the synthesized MOFs were also investigated.

## Results and Discussion

### Synthesis and Structural Analyses of MOFs

**Figure 1:**
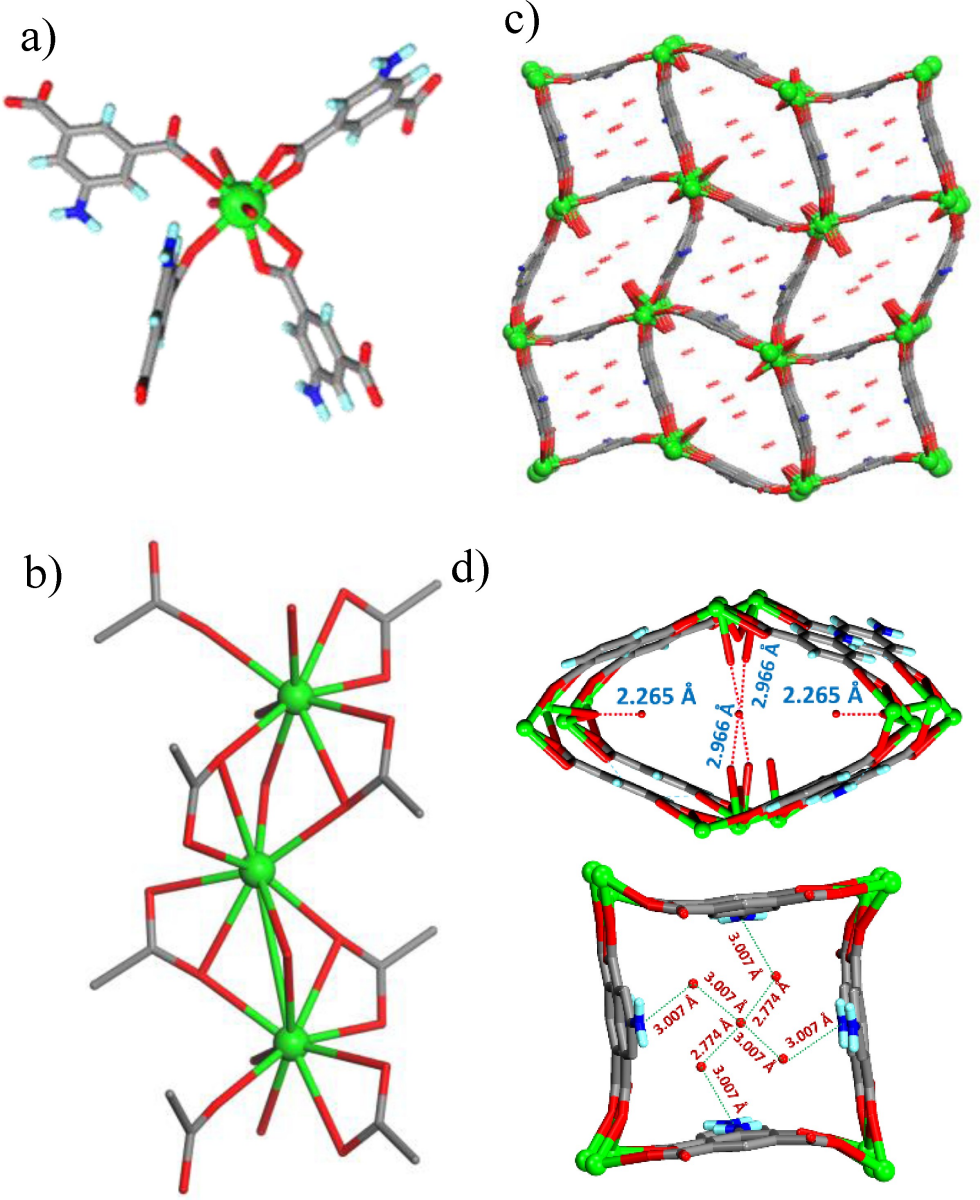
Illustrations for the crystal structure of **Sr‐NH_2_‐MOF**: (a) coordination environment of Sr(II); b) bridging of Sr(II) centers by water and carboxylates to form 1D‐chains (only carboxylates of AmIP units were shown); (c) connecting of 1D‐metal‐crboxylate chains by AmIP units to form 3D‐network containing two types of channels; (d) H‐bonded water molecules in the channels (D…A distances are given).

The solvothermal reaction of **H_2_AmIP** with Sr(NO_3_)_2_ in a DMF‐H_2_O solvent system at 100 °C affords crystals of {[Sr_2_(**AmIP**)_2_(μ_2_‐H_2_O)_2_(H_2_O)] ⋅ 6H_2_O}_n_ (**Sr‐NH_2_‐MOF**), while the reaction of **H_2_BrIP** with Sr(NO_3_)_2_ under the identical conditions resulted in the formation of {[Sr_7_(BrIP)_6_(HBrIP)_2_(DMF)_2_(H_2_O)_6_] ⋅ xSolvent}_n_ (**Sr‐Br‐MOF**). The solvothermal reaction of H_4_OAT and AMT in presence of Zn(NO_3_)_2_.6H_2_O in DMA‐EtOH solvent system resulted in the formation of {[Zn_2_(OAT)(AMT)(EtOH)] ⋅ xSolvent}_n_ (**Zn‐Tz‐MOF**). Pertinent crystallographic details for the three MOFs are given in Table S1. The crystal structure analysis of **Sr‐NH_2_‐MOF** revealed that it crystallized in *P‐4n2* space group and the asymmetric unit is constituted by one unit each of AmIP and Sr(II), a coordinated water molecule and two bridging water molecules, each with half occupancy, and six free water molecules (four with half occupancy and two with full occupancy). The Sr(II) centre exhbits overall nine coordination as it is coordinated by two carboxylates in bidentate fashion, two carboxylate in monodentate fashion, one water molecule and two bridging water molecules. Metal ions form an infinite one‐dimensional chain through bridging water molecules and carboxylate oxygens. These 1D‐chians are connected by the spacer AmIP to form a highly porous three‐dimensional network with two types of channels that are occupied by the water molecules. Uncoordinated water molecules were found to be involved in N−H⋅⋅⋅O hydrogen bonding with the amine group on the isophthalate moiety and forms O−H⋅⋅⋅O hydrogen bond with the neighbouring free water molecule. The calculation by PLATON shows effective desolvated void space is 39 % of the crystal volume (Figure 1).

**Figure 2:**
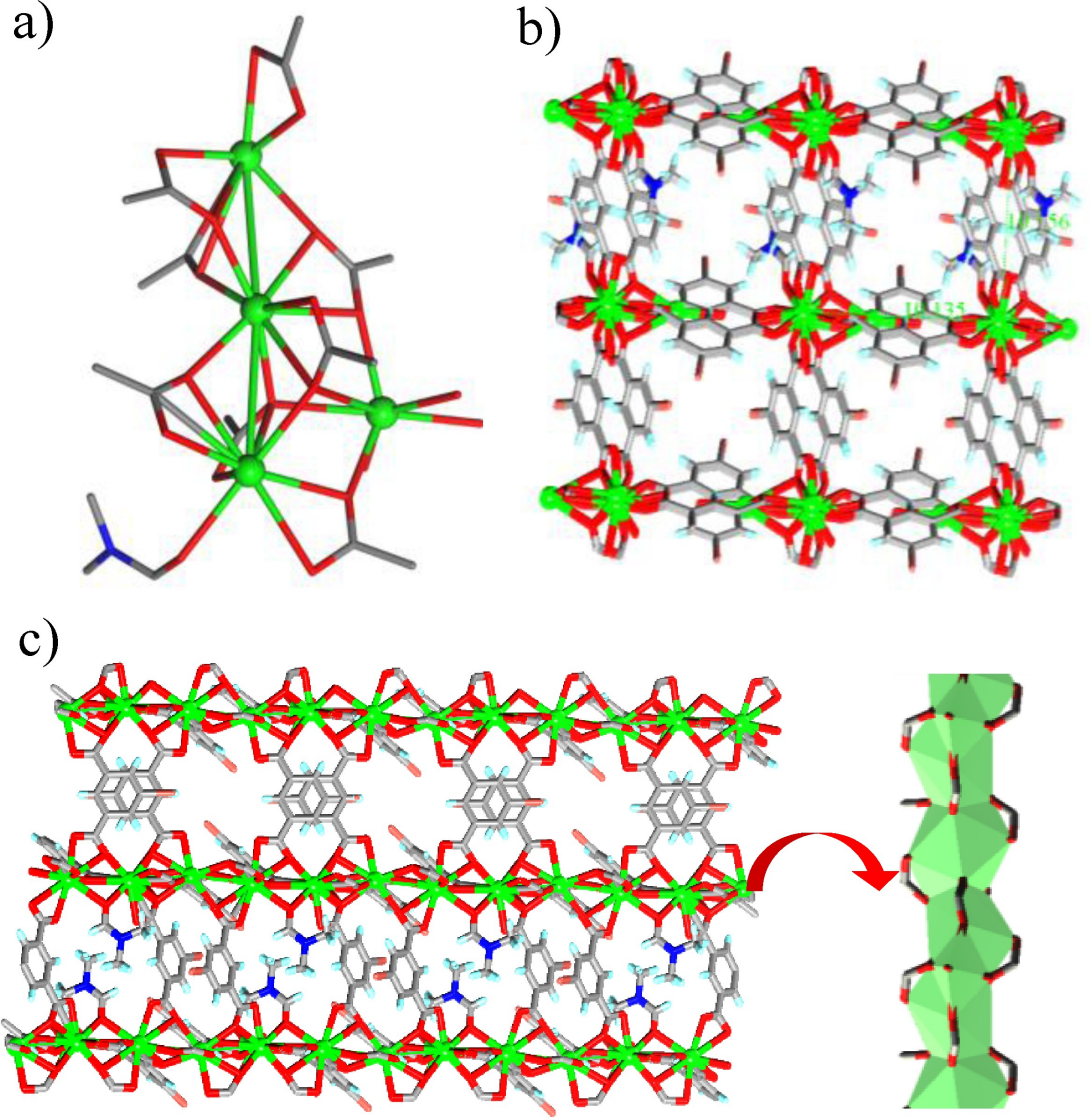
Illustrations for the crystal structure of **Sr‐Br‐MOF**: (a) Sr4 secondary building unit that lead to 1D‐chain of metal‐carboxylate‐metal (only carboxylates of BrIP units were shown) (b) 3D network exhibiting continuous channels that are decorated by Br‐atoms; (c) 3D‐network view in ab‐plane showing 1D metal‐oxygen‐metal chains that are connected by BrIP units to form 3D‐network.

The **Sr‐Br‐MOF** crystallizes in *P‐1* space group and the asymmetric unit contains three **BrIP** and one **HBrIP** units, four crystallographically independent Sr(II) centres of which one with half occupancy, one coordinated DMF and three coordinated water molecules. Out of the four Sr(II) centres two centres exhibit similar coordination geometry with nine O‐atoms: coordinated by three carboxylate in bidentate fashion and two bridging carboxylate O‐atoms from ligands that are connected to neighbouring metal centre and one DMF carbonyl bridging the two centres. The third Sr(II) centre connected to eight O‐atoms: binds to two carboxylates in bidentate fashion and four bridging carboxylate oxygens from the ligands connected to the neighbouring metal centre. The fourth Sr(II) centre exhibits seven coordination as it is coordinated to three water molecules and four carboxylate oxygens from ligand units that are connected to the neighbouring metal centres. In this MOF, three of the metal ions form infinite 1D chain through metal‐oxygen‐metal bonds along *b*‐axis while the fourth one which is coordinated to water connected to the sideways to this linear chain. The linear chains are connected by BrIP spacer to form a 3D network with 14 % solvent accessible void and the pore walls are decorated by Br atoms of isophthalate units (Figure 2).

**Figure 3:**
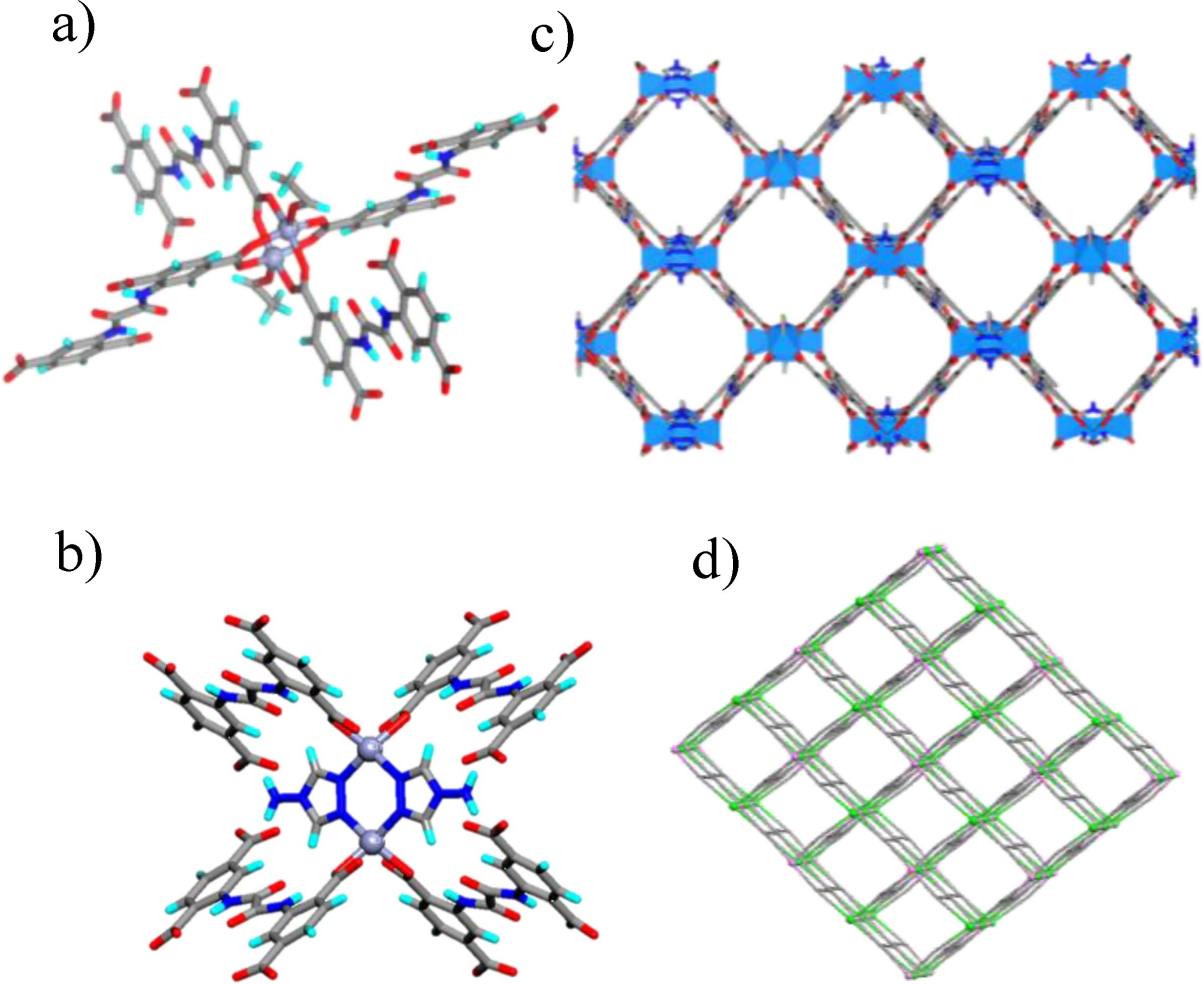
Illustrations for the crystal structure of **Zn‐Tz‐MOF**: bimetallic SBUs a) through the coordination of bridging of carboxylates of OAT; (b) through the coordination of AMT units; c) overall 3D network; d) node connected view of the 3D network.

The single crystals of **Zn‐Tz‐MOF** crystallized in *C2/m* space group and the asymmetric unit is constituted by half unit each of OAT and AMT, two crystallographically independent Zn(II) centres each with half occupancy and one coordinated ethanol molecule with half occupancy. One of the Zn(II) centre was found to be present in a distorted square pyramidal coordination geometry with all the equatorial positions occupied by carboxylate O‐atoms from four ligand units in monodentate fashion and the apical position was occupied by the ethanol oxygen. Such two square pyramidal centres were joined by bridging carboxylates to giving rise to a paddlewheel SBU. The second metal centre forms a distorted octahedral coordination geometry being coordinated by two carboxylate units in bidentate fashion and two triazole nitrogens, two of these centres were bridged via the triazole unit to form a binuclear SBU that act as a four connected node. Such connectivity led to the formation of a porous 3D network and the PLATON calculation shows 59 % solvent accessible void per unit cell (Figure 3). The phase purity of the MOFs was verified by powder X‐ray diffraction (PXRD) studies, which indicate that the diffraction patterns of the samples are consistent with the corresponding calculated ones from the single crystal data.

**Figure 4:**
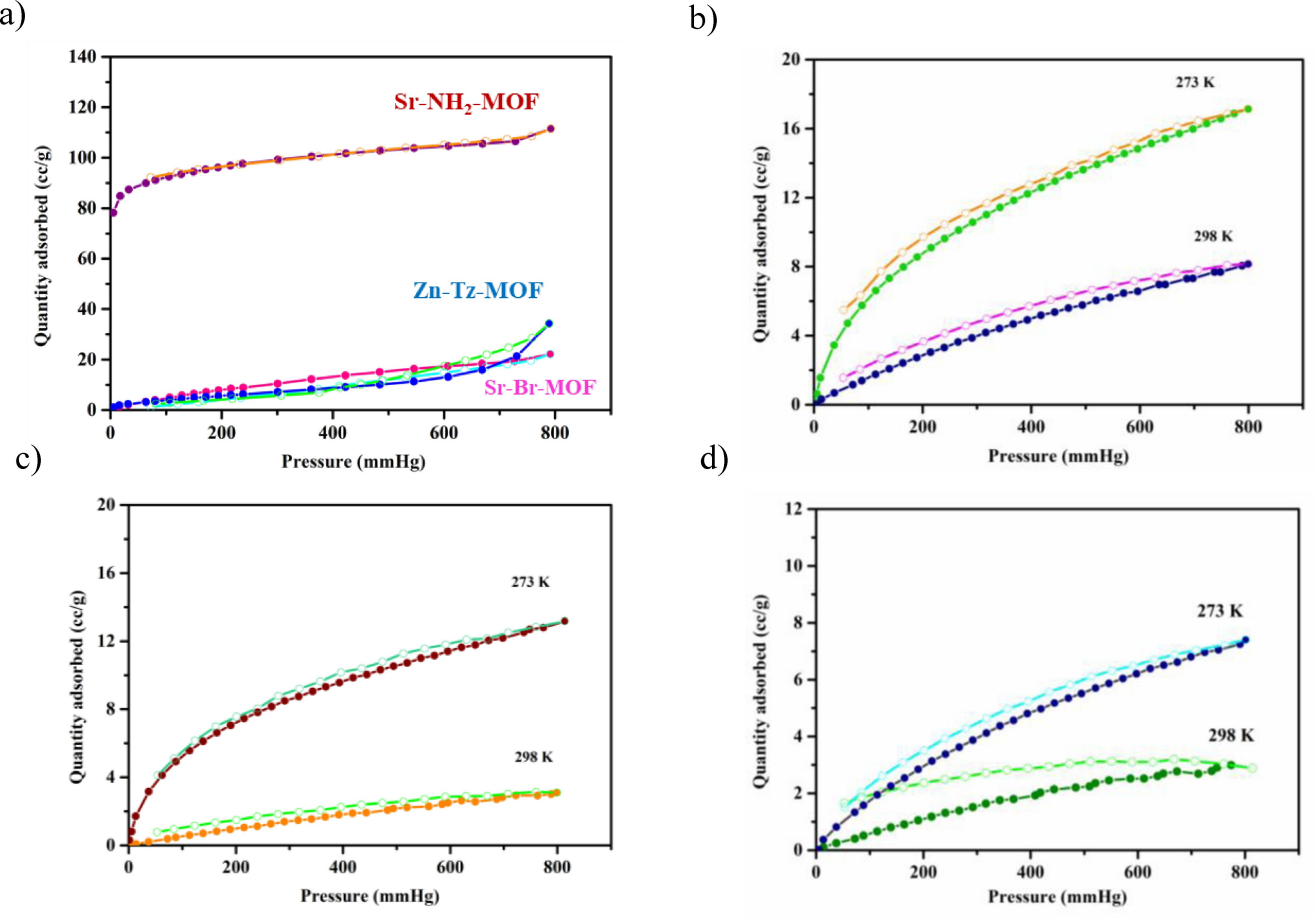
(a) N_2_ adsorption isotherms for the MOFs at 77 K; CO_2_ adsorption isotherms at 273 K and 298 K for (b) **Zn‐Tz‐MOF**, (c) **Sr‐NH_2_‐MOF**, (d) **Sr‐Br‐MOF**.

To assess the permanent porosity of these MOFs, an N_2_ adsorption isotherm at 77 K was measured for the activated sample, which reveals that **Sr‐NH_2_‐MOF** shows a typical type I adsorption behaviour with Brunauer–Emmett–Teller (BET) surface area 289.2 m^2^ g^−1^, while a very low uptake was observed with surface area of 22.1 m^2^ g^−1^ and 39.3 m^2^ g^−1^ (Figure 4a) for **Sr‐Br‐MOF** and **Zn‐Tz‐MOF** respectively.

### Gas Adsorption Studies

The structural features, including permanent porosity, rich NH_2_ groups, and N‐rich triazole units present in these MOFs prompted us to investigate its affinity to CO_2_. The CO_2_ sorption capability of the MOFs were evaluated at 273 K and 298 K. The adsorption curve for CO_2_ indicates that the adsorption capacity of **Sr‐NH_2_‐MOF** and **Zn‐Tz‐MOF** are higher compared to **Sr‐Br‐MOF** (Figure 4b–d). It can be attributed to highly polar channels decorated by amine groups that facilitate stronger interactions with CO_2_ molecules because of the larger quadrupolar moment.

We note here that these adsorption values are comparable with those of some previously reported MOFs. Nevertheless, the CO_2_ philicity of the MOFs indicate that the adsorbed CO_2_ can be utilized to promote the conversion of CO_2_ to cyclic carbonate upon reaction with oxiranes. Further, the inherent CO_2_‐adsorbing property and embedded Lewis acidic metal sites in the framework suggested that the MOFs particularly **Sr‐NH_2_‐MOF** and **Zn‐Tz‐MOF** anticipated to be a highly promising heterogeneous catalyst for CO_2_‐fixation reactions.

### CO_2_ Fixation Reaction

In a typical experiment under solvent‐free conditions, 10 mg MOF as a catalyst along with 5 mol % tetrabutylammonium bromide (TBAB) as a co‐catalyst were immersed into the liquid epoxide (10 mmol) and CO_2_ purged at 1 atm pressure at room temperature and the reaction was monitored for 48 h. The CO_2_ addition reaction with various substrates of unsymmetrical epoxides containing n‐butyl, phenyl, phenyl ether and t‐butyl‐ether substitutions were investigated with all the three MOFs (Table 1). Moreover, a higher conversion percentage was observed for less bulky epoxide such as propylene oxide with all the three MOFs. The large‐sized epoxide substrates have shown lower conversion rates due to their limited diffusion into the channels of the catalyst which inhibits the proximity of catalytic sites for the reactants, thus displaying catalysis in a size‐selective manner.

**Table 1:** Chemical fixation of CO_2_ with epoxides.

Substrate		Sr‐NH2‐MOF			Zn‐Tz‐MOF			Sr‐Br‐MOF		
		Conv. (%)	TON	TOF (h‐1)	Conv. (%)	TON	TOF (h‐1)	Conv. (%)	TON	TOF (h‐1)
1	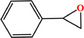	91.7	316	6.59	96.1	322	6.72	36.1	140	2.93
2	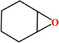	36.1	124	2.58	13.7	46	0.96	12.1	46	0.97
3	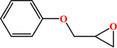	73.4	253	5.27	66.0	221	4.61	61.0	238	4.96
4	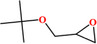	71.4	246	5.12	93.0	312	6.50	43.0	168	3.50
5	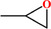	94.6	315	6.58	97.1	325	6.79	68.4	267	5.57
6	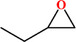	90.9	299	6.24	92.6	310	6.47	54.1	211	4.40
7	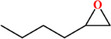	82.6	284	5.93	90.1	302	6.29	62.6	244	5.09

Interestingly, accompanied by the size selectivity of the MOFs there is a significant difference in the performance of the MOFs as **Sr‐NH_2_‐MOF** and **Zn‐Tz‐MOF** performed much better compared to **Sr‐Br‐MOF** with respect to the conversion rates of any given substrate. For example, considering the substrate styrene oxide, the % conversions were 36 %, 91 % and 96 % in presence of the **Sr‐Br‐MOF**, **Sr‐NH_2_‐MOF** and **Zn‐Tz‐MOF** respectively. The trend can be rationalized based on their structural features specifically the amine functional groups present in **Sr‐NH_2_‐MOF** and **Zn‐Tz‐MOF** can interact with the CO_2_ molecules and open metal sites interacting with the oxirane molecules giving rise to a proximity effect facilitating the enhanced conversion rate. While for the other case in presence of bromine substitution it displayed substantial decrease in the percentage conversion due to the absence of any such recognizable interaction with the CO_2_ molecule. Thus, CO_2_ can be captured through the electrostatic binding within the MOF channels and the epoxy ring can be activated through the binding of the epoxide oxygen by metal centres.

**Scheme 2:**
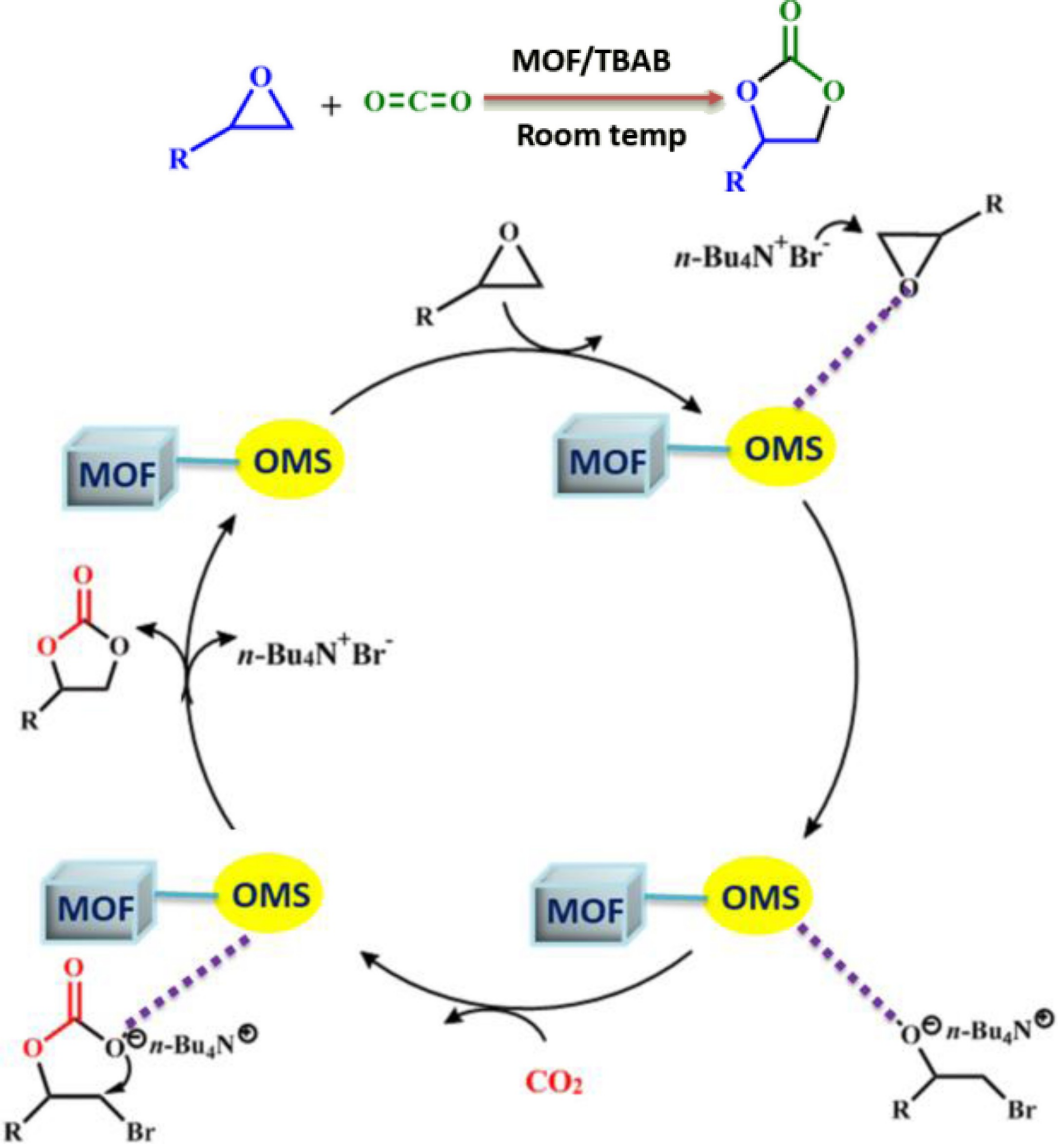
Plausible mechanism for carbon dioxide fixation inside the channels of MOFs. (OMS – Open Metal Sites, represents the Lewis acidic metal nodes and act as active catalytic sites).

Further, a probable mechanism was proposed in coherence with several previously reported works by us and various other groups illustrated in Scheme 2. In this proposed mechanism, at first the epoxide binds with the Lewis acidic metal site in the microporous channel of the MOFs through the epoxide oxygen, this helps activation of the epoxy ring. The less‐hindered carbon atom of the activated epoxide is then attacked by the Br^−^ generated from co‐catalyst TBAB to open ring. Subsequently CO_2_ interacts with the oxygen anion of the opened epoxy ring which is then converted into the corresponding cyclic carbonate through a ring closing step. Another important aspect of a catalyst for practical application is its recyclability. The recyclability of all the MOFs were studied by reusing the catalyst three times successively for the cycloaddition reaction of CO_2_ with styrene oxide, where the catalytic activity remained almost same for those three successive cycles. After each cycle, MOFs were recovered through centrifugation, followed by **Sr‐NH_2_‐MOF** and **Sr‐Br‐MOF** were washed with acetone while **Zn‐Tz‐MOF** was washed with ethanol and dried in vacuum to use it for next catalytic cycle. The PXRD patterns of respective MOFs before and after catalysis remained the same, revealing its structural integrity after the catalytic experiments (Section S3).

## Conclusions

In conclusion, three new crystalline and porous MOFs were synthesized and explored for their catalytic activity towards CO_2_ addition reactions to epoxides. The **Sr‐NH_2_‐MOF** and **Zn‐Tz‐MOF** have exhibited very similar catalytic activity despite of having differences in their metal atoms, ligands and porosity. From these studies, it can be concluded that the alkaline earth metal ion Sr(II) is as good as first row transition metal ion Zr(II) atom in catalyzing CO_2_ addition reaction to epoxides. The inherent CO_2_ adsorption ability, and higher catalytic activity was attributed to well‐defined pores decorated with lewis basic amine groups and exposed Lewis‐acid metal sites enable the MOF to catalyze CO_2_ fixation of epoxides with size‐selectivity and high efficiency under ambient conditions in a one‐pot reaction. The role of amino group in yielding higher percentage of CO_2_ conversions is apparent from comparatively low catalytic activity exhibited Sr‐Br‐MOF. In addition, the MOFs were found to exhibit excellent recyclability by simple filtration and washing and reused multiple times without any considerable loss of activity. Thus the results demonstrate that utilization of alkaline earth metal ions containing large ionic radius together with amine functionalized ligands is an effective strategy to fabricate a stable and porous MOFs for the capture of CO_2_ and chemical conversion of CO_2_ molecules for value‐added products with high efficiency.

## Experimental Section

All the chemicals such as metal salts, aminotriazole, aminoisophthalic acid, bromoisophthalic acid were purchased from Alfa Aesar, and solvents such as DMF, EtOH, DMA were purchased from local chemical suppliers; various epoxides were purchased from Sigma‐Aldrich and used without further purification. Perkin‐Elmer, UATR Two spectrometer was used to record the IR spectra. Powder X‐ray diffraction (PXRD) data were recorded with a Bruker D8‐advance diffractometer at room temperature. ^1^H were recorded with BRUKER‐AC 400 MHz NMR instrument.

### Synthesis of MOFs

#### Sr‐NH_2_‐MOF

(0.018 g, 0.1 mmol) 5‐aminoisophthalic acid was mixed with Sr(NO_3_)_2_ (0.021 g, 0.1 mmol) in N, N‐dimethylformamide (DMF, 4 mL) with the addition of 1 mL water. Then the mixture was taken into a tightly capped 15 mL pyrex tube and then it was heated at 100 °C for 24 hrs. The resulted brown block shaped crystals were collected after cooling it down to room temperature. After that the crystals were washed with DMF for several times. (Yield: ~79 %)

#### Sr‐Br‐MOF

(0.024 g, 0.1 mmol) 5‐bromoisophthalic acid was mixed with Sr(NO_3_)_2_ (0.021 g, 0.1 mmol) in N, N‐dimethylformamide (DMF, 4 mL) with the addition of 1 mL water. Then the mixture was taken into a tightly capped 15 mL pyrex tube and then it was heated at 100 °C for 24 hrs. The resulted white block shaped crystals were collected after cooling it down to room temperature and washed with DMF for several times. (Yield: ~68 %)

#### Zn‐Tz‐MOF

(0.021 g, 0.05 mmol) **H_4_OAT** and (0.018 g, 0.2 mmol) **AMT** was mixed with Zn(NO_3_)_2_.6H_2_O (0.029 g, 0.1 mmol) in N,N‐dimethylacetamide (DMA, 4 mL) with the addition of 1 mL ethanol. Then the mixture was taken into a tightly capped 15 mL pyrex tube and then it was heated at 100 °C for 24 hrs. The resulted white block shaped crystals were collected after cooling it down to room temperature and washed with DMA. (Yield: ~74 %)

### Crystal Structure Determination

All the single crystal data were collected on a Bruker‐D8‐Venture X‐ray diffractometer that uses graphite monochromated Mo Kα radiation (λ=0.71073 Å) by the hemisphere method. The structures were solved by direct methods and refined by least‐squares methods on F^2^ using SHELX‐2014.^[47]^ Non‐hydrogen atoms were refined anisotropically, and hydrogen atoms were fixed at calculated positions and refined using a riding model. In all three MOFs, the solvent molecules are found to be disordered. Therefore, the final refinement was carried out by removing the solvents and using PLATON squeeze option.

## Conflict of Interests

The authors declare no conflict of interest.

1

## Supporting information

As a service to our authors and readers, this journal provides supporting information supplied by the authors. Such materials are peer reviewed and may be re‐organized for online delivery, but are not copy‐edited or typeset. Technical support issues arising from supporting information (other than missing files) should be addressed to the authors.

Supporting Information

## Data Availability

The data that support the findings of this study are available in the supplementary material of this article.
