# Supplementary material for: Amine Functionalization of Channels of Metal‐Organic Frameworks for Effective Chemical Fixation of Carbon Dioxide: A Comparative Study with Three Newly Designed Porous Networks
Source: ChemistryOpen. 2024 May 13;13(9):e202400110. doi: 10.1002/open.202400110 (PMC11633332; doi:10.1002/open.202400110)
Supplement: Supplementary file 1 — Supporting Information [file OPEN-13-e202400110-s001.pdf]

ChemistryOpen

Supporting Information

Amine Functionalization of Channels of Metal-Organic Frameworks for Effective Chemical Fixation of Carbon Dioxide: A Comparative Study with Three Newly Designed Porous Networks

Rajib Moi, Swati Bedi, and Kumar Biradha*

Supporting Information

Amine Functionalization of Channels of Metal-Organic Frameworks for Effective Chemical Fixation of Carbon Dioxide: A Comparative Study with Three Newly Designed Porous Networks

Rajib Moi, Swati Bedi, and Kumar Biradha*

E-mail: kbiradha@chem.iitkgp.ernet.in

Contents

1. General aspects
2. Synthesis of MOF
3. Crystal structure determination
4. PXRD
5. FTIR
6. Details of Gas adsorption studies
7. Details of Catalytic studies
8. NMR Spectra
9. References

General:

All of the chemicals such as metal salts, aminotriazole, aminoisophthalic acid, bromoisophthalic acid was purchased from Alfa Aesar, and solvents such as DMF, EtOH, DMA were purchased from local chemical suppliers; various epoxides were purchased from Sigma-Aldrich and used without further purification. Parkin-Elmer, UATR Two spectrometer was used to record the IR spectra. Powder X-ray diffraction (PXRD) data were recorded with a Bruker D8-advance diffractometer at room temperature. ^{1}H-NMR were recorded with BRUKER-AC 400 MHz NMR instrument.

Section S1: Synthesis of MOFs

Sr-NH_2-MOF

(0.018 g, 0.1 mmol) 5-aminoisophthalic acid was mixed with $Sr(NO_3)_2$ (0.021 g, 0.1 mmol) in N,N-dimethylformamide (DMF, 4 mL) with the addition of 1 mL water. Then the mixture was taken into a tightly capped 15 mL pyrex tube and then it was heated at 100˚C for 24 hrs. The resulted brown block shaped crystals were collected after cooling it down to room temperature. After that the crystals were washed with DMF for several times. (Yield: ~79%)

Sr-Br-MOF

(0.024 g, 0.1 mmol) 5-bromoisophthalic acid was mixed with $Sr(NO_3)_2$ (0.021 g, 0.1 mmol) in N,N-dimethylformamide (DMF, 4 mL) with the addition of 1 mL water. Then the mixture was taken into a tightly capped 15 mL pyrex tube and then it was heated at 100˚C for 24 hrs. The resulted white block shaped crystals were collected after cooling it down to room temperature and washed with DMF for several times. (Yield: ~68 %)

Zn-Tz-MOF

(0.021 g, 0.05 mmol) **H_4OAT** and (0.018 g, 0.2 mmol) **AMT** was mixed with $Zn(NO_3)_2.6H_2O$ (0.029 g, 0.1 mmol) in N,N-dimethylacetamide (DMA, 4 mL) with the addition of 1 mL ethanol. Then the mixture was taken into a tightly capped 15 mL pyrex tube and then it was heated at 100˚C for 24 hrs. The resulted white block shaped crystals were collected after cooling it down to room temperature and washed with DMA. (Yield: ~74 %)

Section S2: Crystal Structure Determination

All the single crystal data were collected on a Bruker-APEX-II CCD X-ray diffractometer that uses graphite monochromated Mo Kα radiation (λ = 0.71073 Å) by the hemisphere method. The structures were solved by direct methods and refined by least-squares methods on F^2 using SHELX-2014.[S1] Non-hydrogen atoms were refined anisotropically, and hydrogen atoms were fixed at calculated positions and refined using a riding model.

Section S3: PXRD Patterns

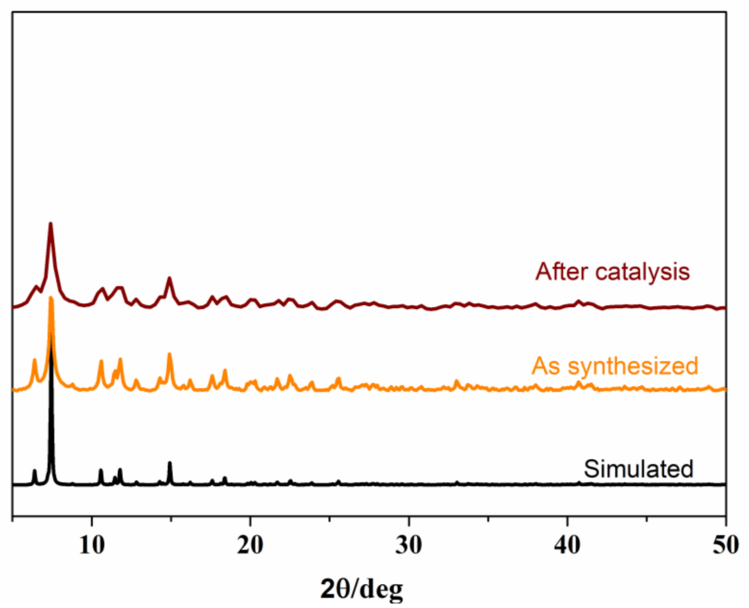

Figure S1. PXRD patterns of the **Zn-Tz-MOF** before and after catalysis.

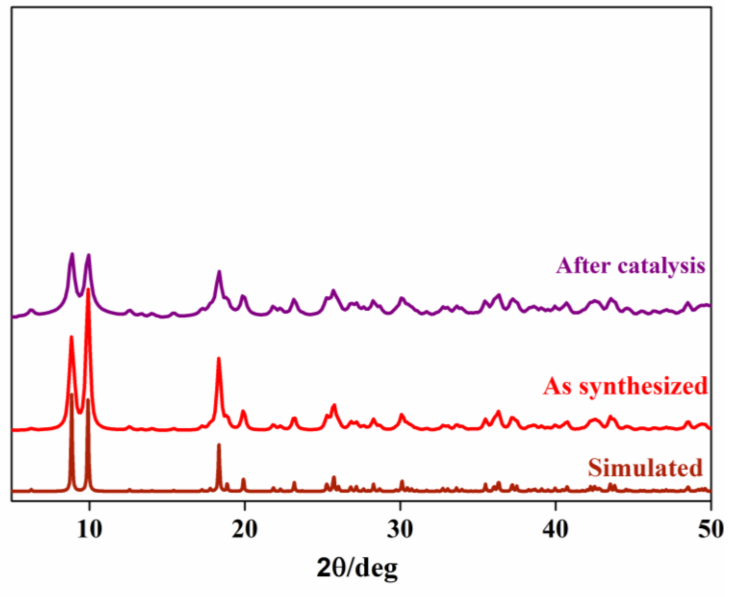

Figure S2. PXRD patterns of the **Sr-NH$_2$-MOF** before and after catalysis.

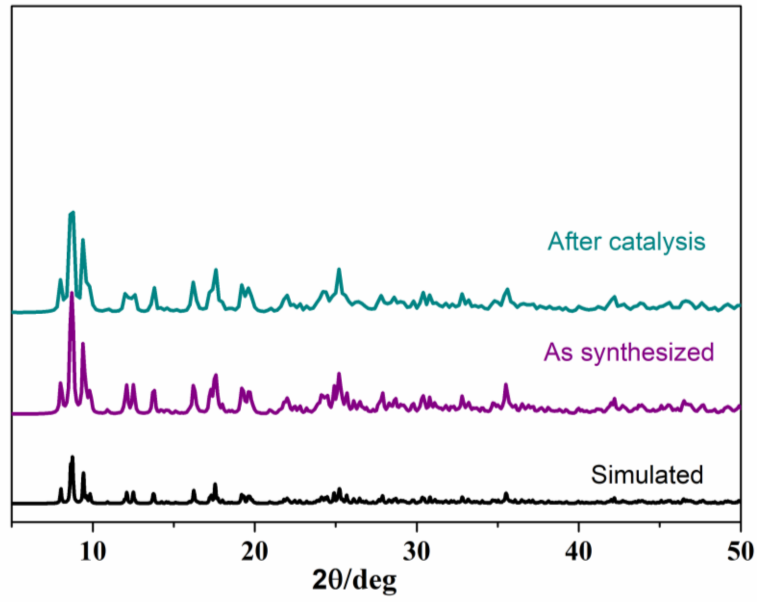

Figure S3. PXRD patterns of the **Sr-Br-MOF** before and after catalysis.

Section S4: FTIR

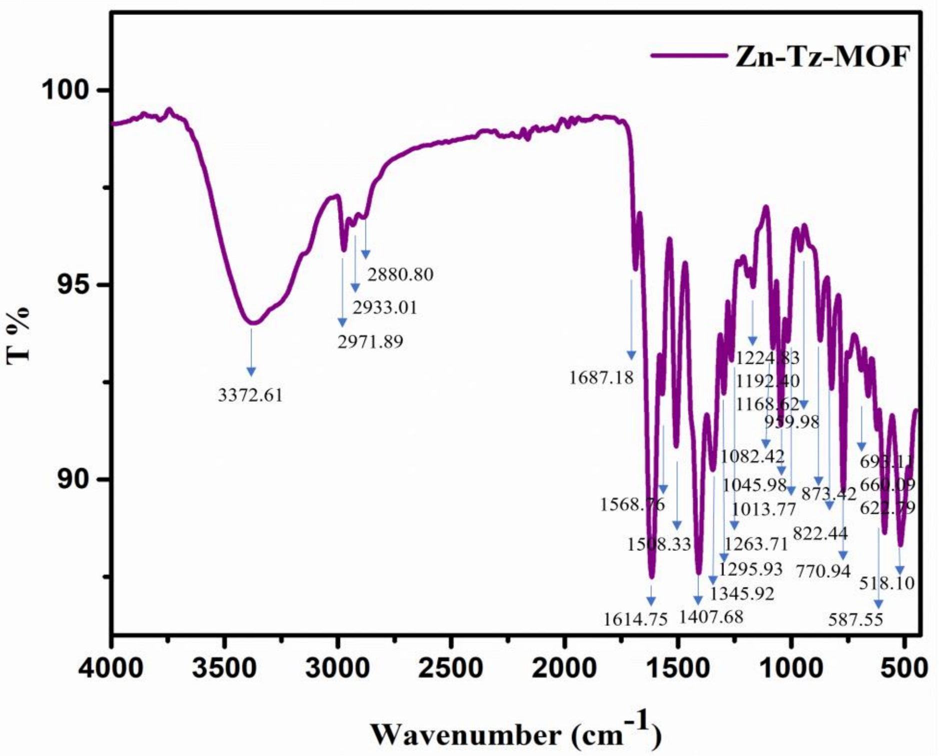

Figure S4. IR spectra of as synthesized **Zn-Tz-MOF**.

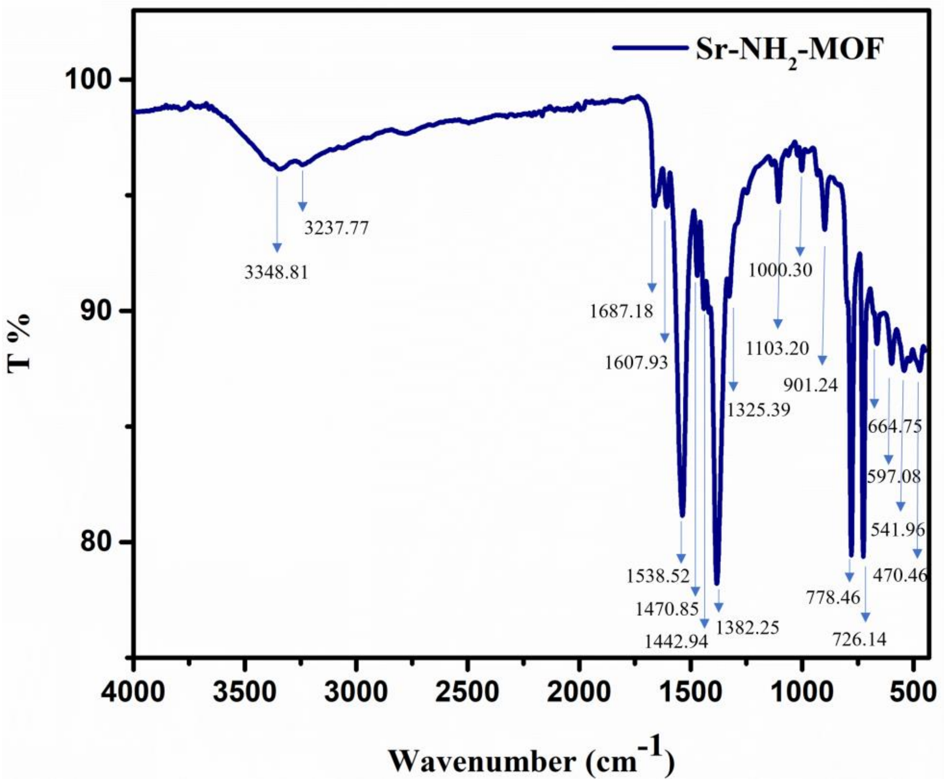

Figure S5. IR spectra of as synthesized **Sr-NH$_2$-MOF**.

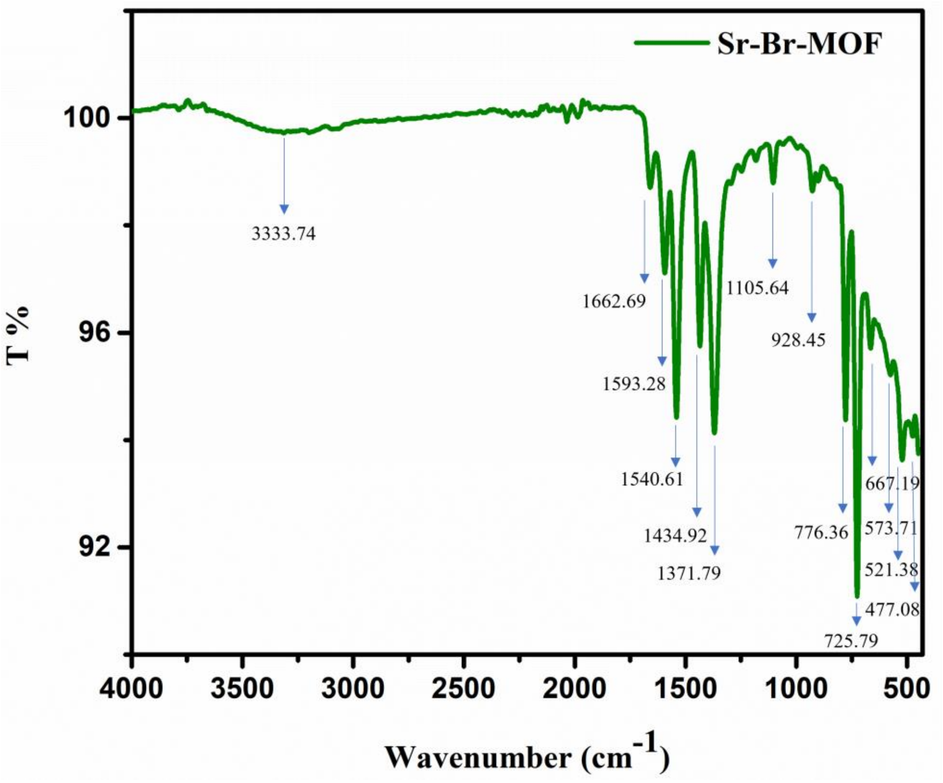

Figure S6. IR spectra of as synthesized **Sr-Br-MOF**.

Section S5: Gas Adsorption Studies

Quantachrome Autosorb-iQ Instruments was used for the low pressure gas sorption (N_2) measurements. The as synthesized MOF crystals were exchanged with acetone/Methanol for seven days by changing the solvent twice in a day. After that the solvent exchanged MOF crystals were taken into a cell in order to activate it before the measurement. The activation was done at 100°C by using the “outgasser” function for 10 hrs. The Brunauer−Emmett−Teller (BET) surface area was calculated from the N_2 sorption isotherms at 77 K. The CO_2 sorption measurement were performed following the same activation procedure using 3Flex–Surface Characterization Analyzer Instrument (Micromeritics).

Section S6: Catalytic Studies

General procedure for CO_2 fixation to epoxides:

In a typical reaction, cycloaddition of the epoxide (10 mmol) was carried out with carbon dioxide purged through balloon at 1 bar pressure using the MOFs (0.3 mol %) in presence of tetrabutylammonium bromide (TBAB, 5 mol%) as a cocatalyst at RT under solvent free condition for 48 hours. The % conversion of the products were calculated based on the 1H NMR analysis and the products were also confirmed by GCMS analysis.

To check the reusability of the MOFs, the reaction mixture was filtered to recover the catalyst after each cycle of the catalysis. Then the recovered catalyst was washed with acetone. Then it was dried under vacuum and used for the next catalytic cycle. The catalytic reaction was carried out for successive three cycles with propylene oxide as substrate for all the three MOFs.

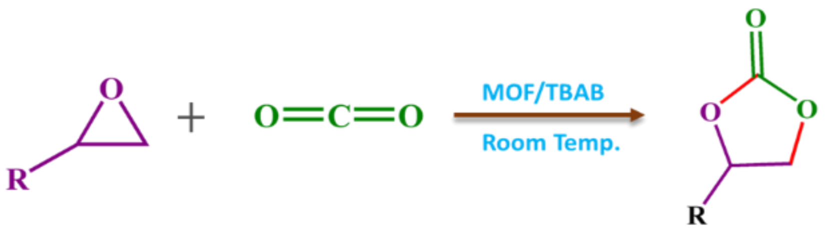

Conversion (%) = $[^1H_b/(^1H_a + {}^1H_b)]$ X 100%

Calculations of TON and TOF:

$$\textbf{TON} = \frac{\text{epoxide (mmol)}}{\text{catalyst (mmol)}} \times \frac{\text{conversion in \%}}{100}$$

$$\textbf{TOF (h}^{-1}\textbf{)} = \frac{\text{TON}}{\text{reaction carried out (hour)}}$$

Plausible mechanism for the Catalysis-

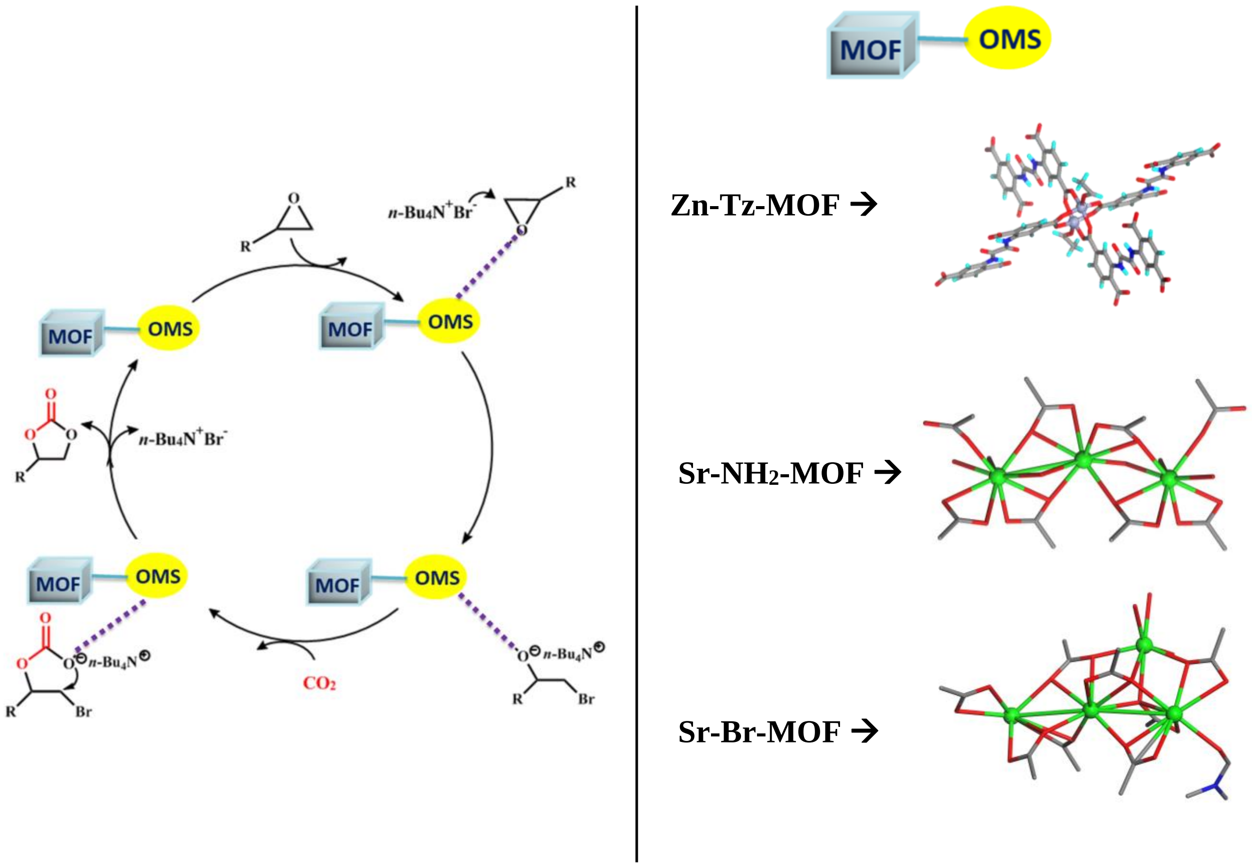

Figure S7. Plausible mechanism for carbon dioxide fixation inside the channels of MOFs (the possible OMS of different MOFs are shown on the right side).

Section S7: Control Experiment for the Catalysis-

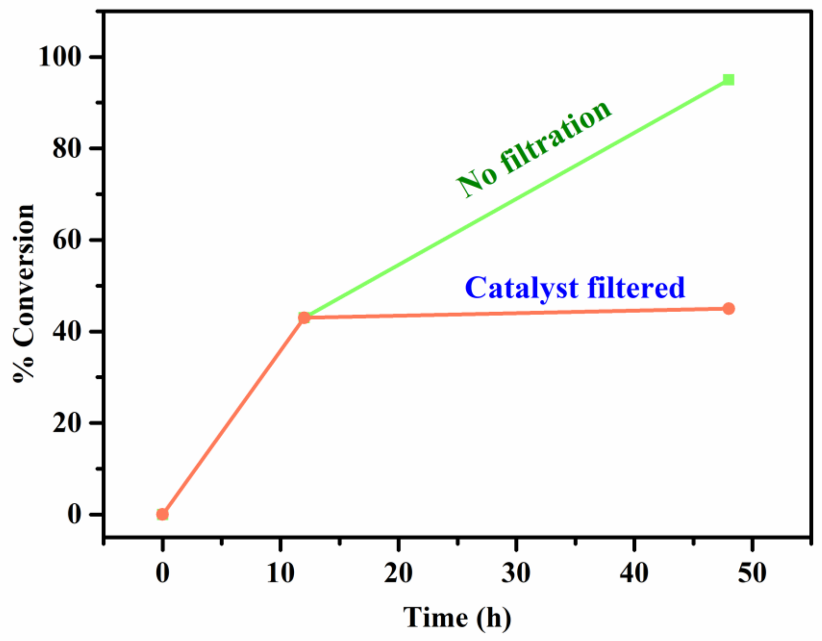

Figure S8. Filtration test (substrate: styrene oxide): with Zn-Tz-MOF; catalyst was filtered after 12 hour (red).

Section S8: Recyclability experiment for the MOFs catalyst-

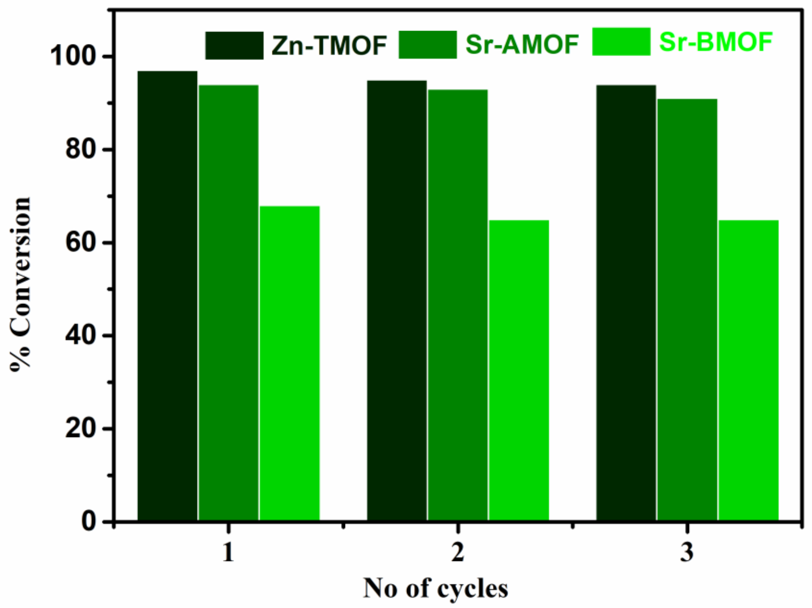

Figure S9. Recyclability experiment for the MOFs for chemical conversion of CO_2.

Section S9: NMR Spectra (^{1}H-NMR) -

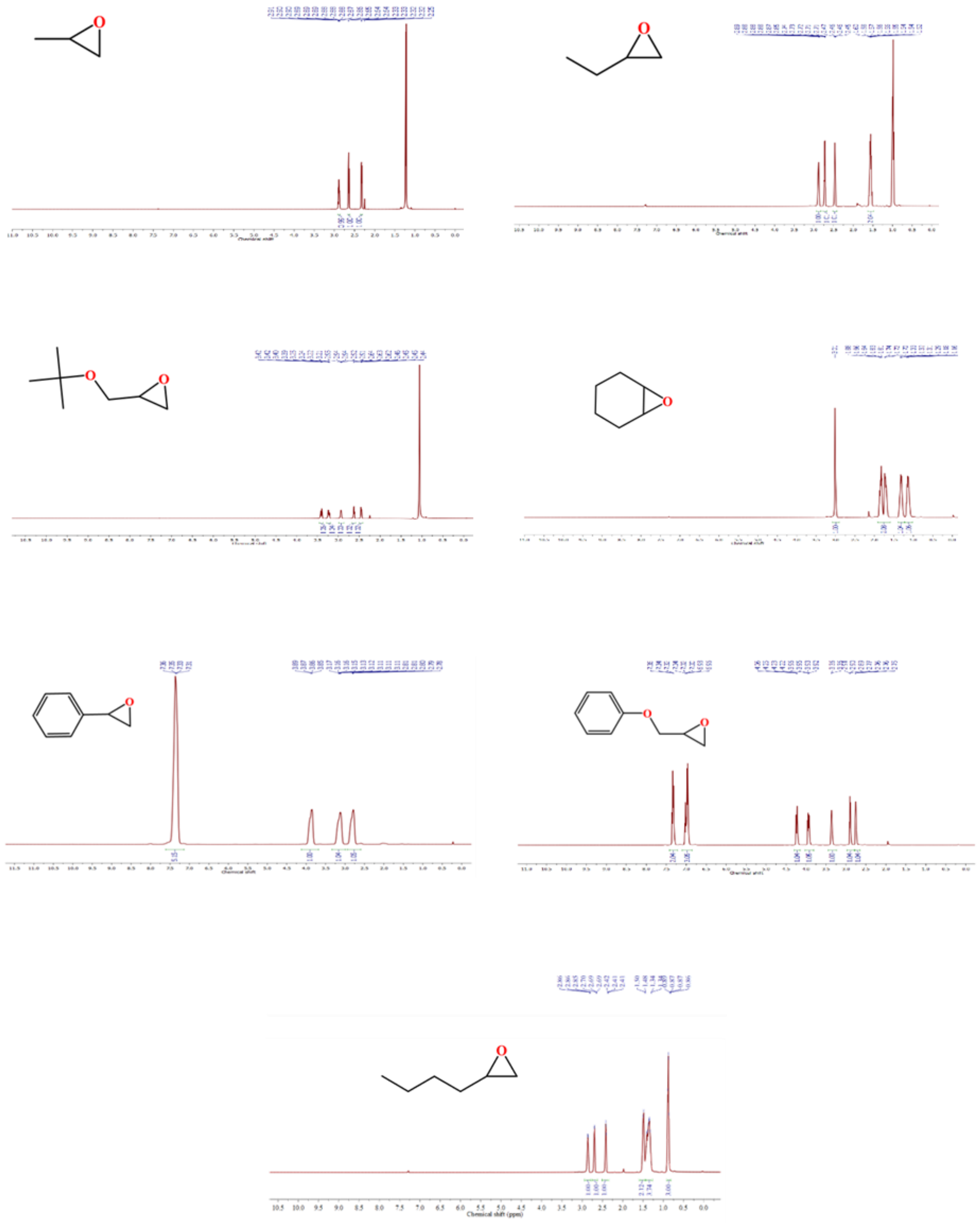

Figure S10. ^{1}H NMR spectra of different type of epoxides (400 MHz, $CDCl_3$).

NMR Spectra (^{1}H-NMR) (Products for cycloaddition of CO_2 to epoxides)-

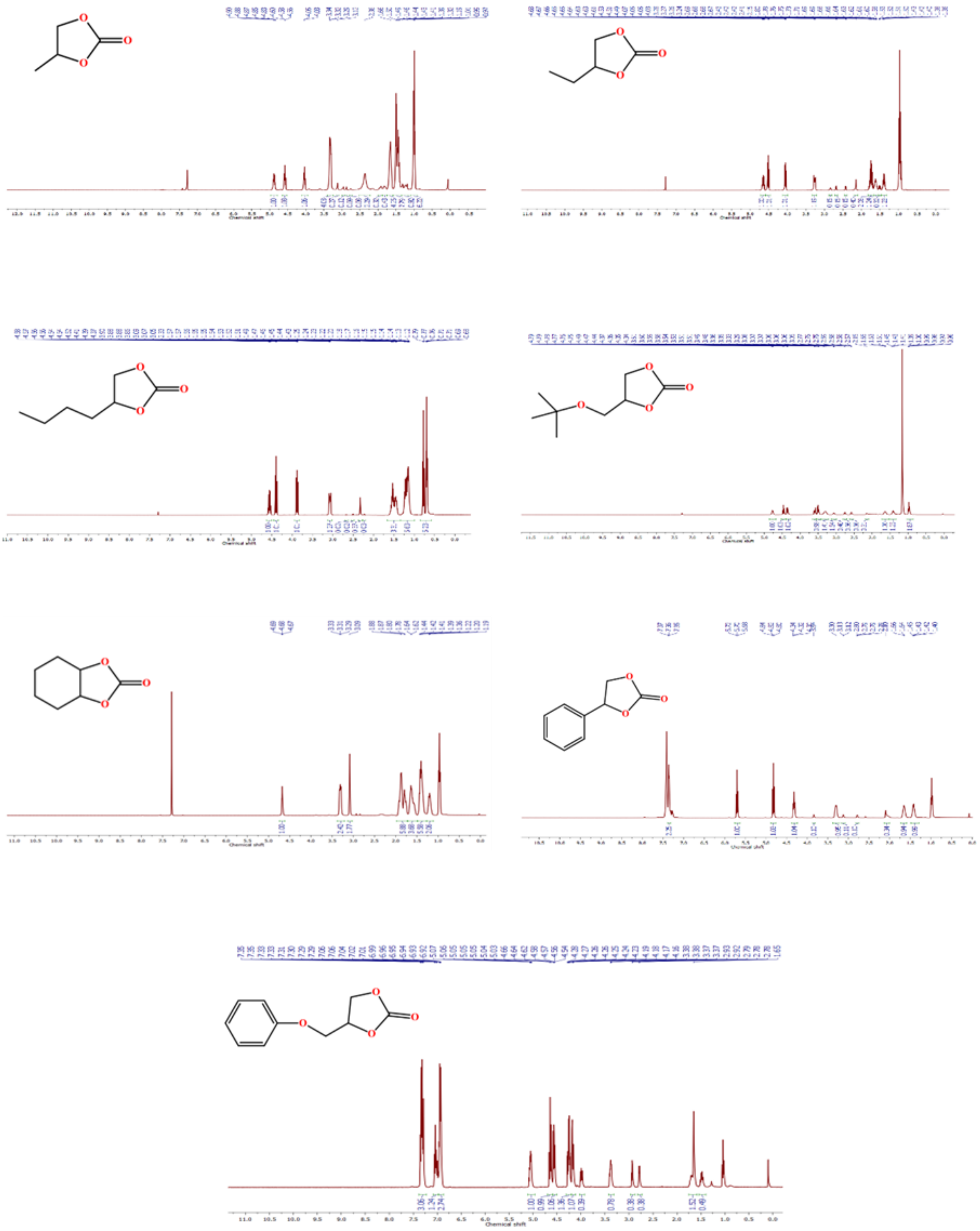

Figure S11. ^{1}H NMR (400 MHz, $CDCl_3$) spectra of the neat reaction mixture containing the cycloaddition product (catalyst used: **Sr-NH_2-MOF**).

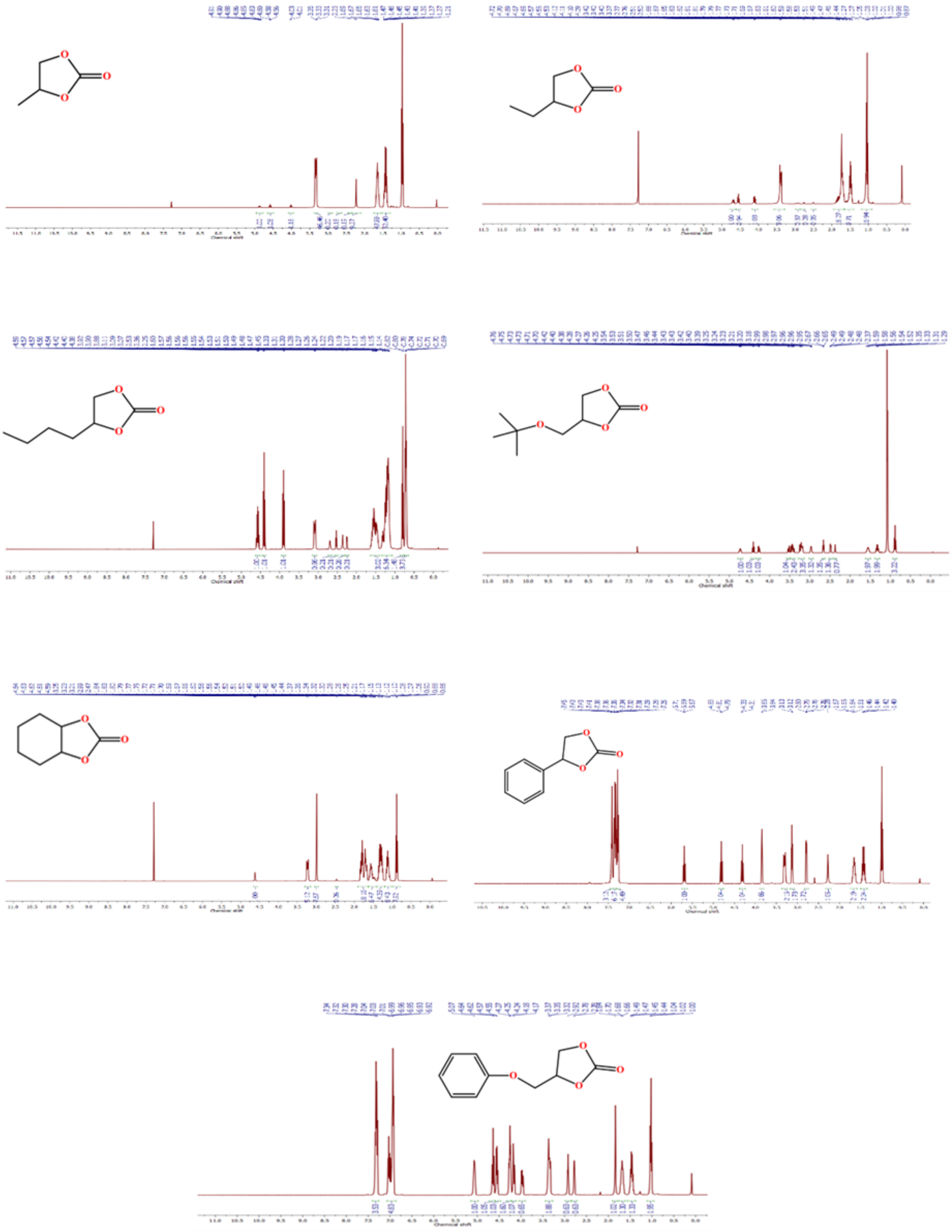

Figure S12. ^{1}H NMR (400 MHz, $CDCl_3$) spectra of the neat reaction mixture containing the cycloaddition product (catalyst used: **Sr-Br-MOF**).

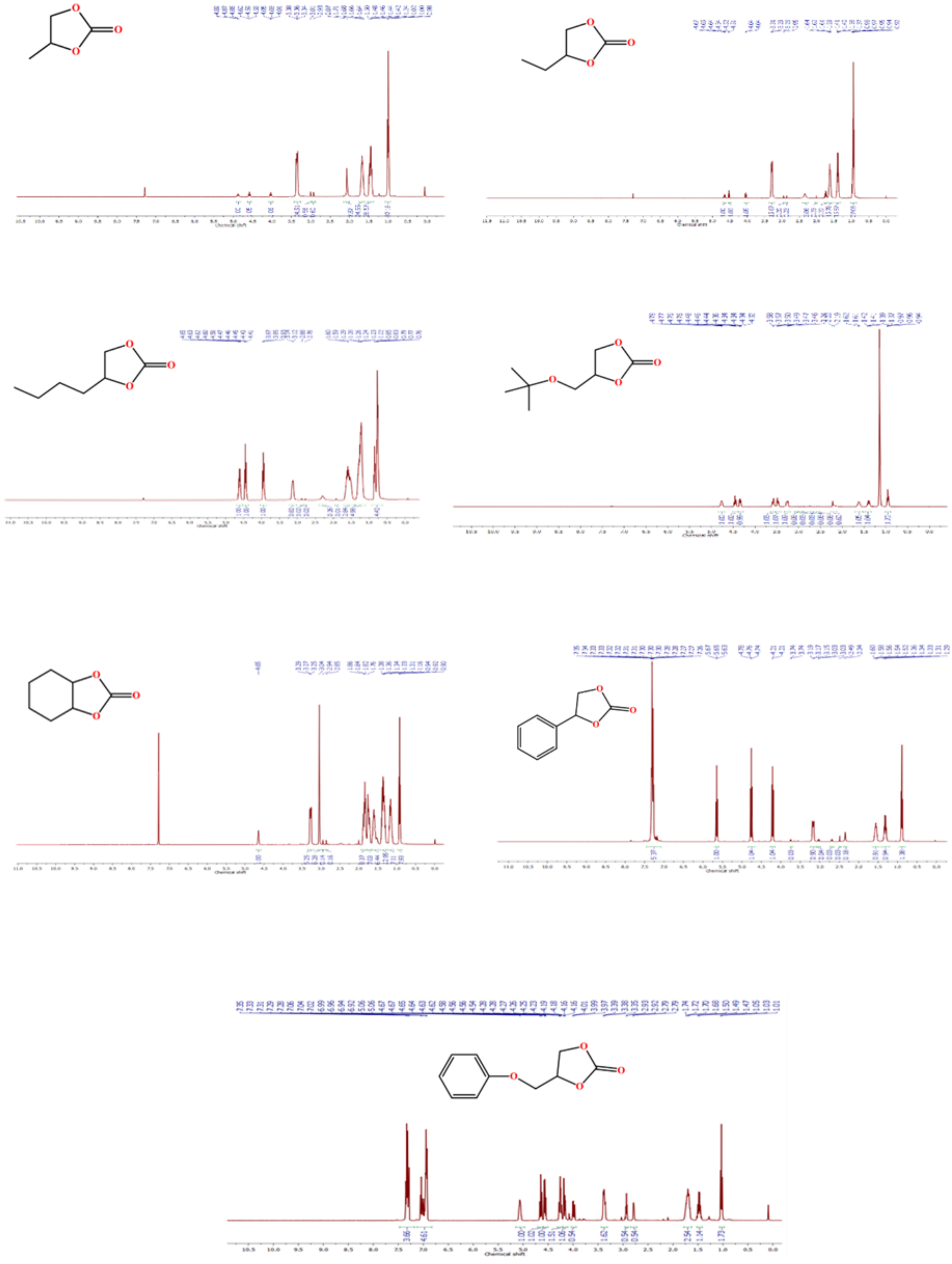

Figure S13. ^{1}H NMR (400 MHz, $CDCl_3$) spectra of the neat reaction mixture containing the cycloaddition product (catalyst used: **Zn-Tz-MOF**).

Table S1: Crystallographic parameters for the MOFs.

MOF	**Sr-NH2-MOF**	**Sr-Br-MOF**	**Zn-Tz-MOF**
CCDC			
Formula	$Sr_2C_{16}H_{22}N_2O_{14}$	$Sr_7C_{70}H_{52}Br_8N_2O_{40}$	$Zn_2C_{22}H_{18}N_6O_{11}$
MW	641.59	2813.75	673.16
Temperature (K)	120(2)	150(2)	156(2)
λ (Å)	0.71073	0.71073	0.71073
Crystal system	Tetragonal	Triclinic	Monoclinic
Space group	*P-4n2*	*P-1*	*C2/m*
a (Å)	19.9429(1)	11.0199(9)	20.1449(1)
b (Å)	19.9429(1)	11.9264(9)	17.8963(1)
c (Å)	7.5050(5)	20.4245(1)	16.633(2)
α (deg)	90	81.588(4)	90
β (deg)	90	85.183(4)	123.971(4)
γ (deg)	90	68.313(4)	90
V (Å3)	2984.9(4)	2466.2(3)	4972.9(8)
Z	4	1	4
d (g/cm3)	1.428	1.895	0.899
R1 (I>2σ(I))	0.0433	0.0692	0.0700

Table S2: Conversion efficiency for styrene oxide to styrene carbonate by some reported MOFs

	**MOF**	**Conv %**	**TOF**	**Ref**
1	Zn-MOF-184	96	5.1	*Inorg. Chem.* **2020**, 59, 16747–16759
2	Mg-MOF-184	66	3.8	*Inorg. Chem.* **2020**, 59, 16747–16759
3	Zn-MOF-74	88	4.6	*Inorg. Chem.* **2020**, 59, 16747–16759
4	Mg-MOF-74	27	1.6	*Inorg. Chem.* **2020**, 59, 16747–16759
5	MOF-177	56	2.7	*Inorg. Chem.* **2020**, 59, 16747–16759
6	MOF-508	77	1.6	*Inorg. Chem.* **2020**, 59, 16747–16759
7	Sr-NH_2-MOF	91	6.5	This Work
8	Sr-Br-MOF	36	2.9	This Work
9	Zn-Tz-MOF	96	6.7	This Work

References

S1. Sheldrick, G. M. SHELXL-2014; University of Göttingen and Bruker AXS: Karlsruhe, Germany, **2014**
